# Hypericin-photodynamic therapy inhibits the growth of adult T-cell leukemia cells through induction of apoptosis and suppression of viral transcription

**DOI:** 10.1186/s12977-019-0467-0

**Published:** 2019-02-19

**Authors:** Lingling Xu, Xueqing Zhang, Wenzhao Cheng, Yong Wang, Kaining Yi, Zhilong Wang, Yiling Zhang, Linxiang Shao, Tiejun Zhao

**Affiliations:** 1 College of Chemistry and Life Sciences, Zhejiang Normal University, 688 Yingbin Road, Jinhua, 321004 Zhejiang China; 2 Biomedical Department, Huaqiao University, Quanzhou, China

**Keywords:** Hypericin, Photodynamic therapy, HTLV-1, ATL, Apoptosis

## Abstract

**Background:**

Adult T-cell leukemia (ATL) is an aggressive neoplasm caused by human T-cell leukemia virus type 1 (HTLV-1). ATL carries a poor prognosis due to chemotherapy resistance. Thus, it is urgent to develop new treatment strategies. Hypericin (HY) is a new-type of photosensitizer in the context of photodynamic therapy (PDT) due to its excellent photosensitizing properties and anti-tumor activities.

**Results:**

In the present study, we investigated the efficacy of hypericin in ATL cells. Clinically achievable concentrations of hypericin in association with PDT induced the inhibition of cell proliferation in ATL cell lines with minimal effect on peripheral blood CD4^+^ T lymphocytes. Moreover, hypericin-PDT treatment caused apoptosis and G2/M phase cell cycle arrest in leukemic cells. Western blot analyses revealed that hypericin-PDT treatment resulted in downregulation of Bcl-2 and enhanced the expression of Bad, cytochrome C, and AIF. Cleavage of caspases-3/-7/-9/-8, Bid, and PARP was increased in hypericin-PDT-treated ATL cells. In a luciferase assay, hypericin-PDT treatment was able to activate the promoter activity of Bax and p53, resulting in enhanced expression of Bax and p53 proteins. Finally, hypericin-PDT treatment suppressed the expression of viral protein HBZ and Tax by blocking the promoter activity via HTLV-1 5′LTR and 3′LTR.

**Conclusions:**

Our results revealed that hypericin-PDT is highly effective against ATL cells by induction of apoptosis and suppression of viral transcription. These studies highlight the promising use of hypericin-PDT as a targeted therapy for ATL.

**Electronic supplementary material:**

The online version of this article (10.1186/s12977-019-0467-0) contains supplementary material, which is available to authorized users.

## Background

Adult T-cell leukemia (ATL) is an aggressive malignancy of CD4^+^ T lymphocytes in which the human T-cell leukemia virus type 1 (HTLV-1) has been recognized as the etiologic agent [1]. HTLV-1 encodes two oncogenic factors, Tax and human T-cell leukemia virus type 1 bZIP factor (HBZ), in the sense and antisense strands of provirus, respectively, and these two factors are thought to play indispensable roles in the oncogenic process of ATL [2, 3].

ATL develops after a long latency period, and the prognosis of ATL patients is still poor due to its resistance to chemotherapy and immunodeficiency [4–6]. Some reports have shown the efficiency of combination therapy of zidovudine and interferon alpha (AZT/IFN) for treatment of ATL, even though little is known about underlying mechanism of AZT/IFN therapy [7–9]. In addition, allogeneic stem cell transplantation is effective in some ATL cases, with remarkable reductions in proviral load to undetectable levels, suggesting that enhancement of the immune response to HTLV-1 is a possible strategy for treatment of HTLV-1-associated human diseases [10, 11]. Unfortunately, most of the patients relapse, stressing the need for alternative or complementary therapies.

Photodynamic therapy (PDT) is a clinical example in which optical illumination selectively activates light-sensitive drugs, termed photosensitizers, and destroys malignant cells with only mild side effects associated with systemic treatments, such as chemotherapy [12]. The use of PDT as an anti-cancer therapy has gained momentum over the past decade, with a number of reports revealing its efficacy with respect to cancers of the head and neck, brain, lung, pancreas, intraperitoneal cavity, breast, prostate and skin [13–16]. Known mechanisms of anti-tumor activity of PDT have been extensively studied. In addition to destroying tumor tissue by a process that can produce cellular necrosis and apoptosis, PDT produces acute inflammation and attracts leukocytes to the treated tumors [17, 18].

Hypericin (HY), a natural polycyclic quinone, is mainly extracted from St John’s Wort (*Hypericum perforatum* L.) [19]. In the context of PDT, hypericin is a promising photosensitizer due to its excellent photosensitizing properties, tumoritropic characteristics, low cytotoxicity, and antiviral activity [19–21]. Hypericin-mediated PDT has gained increasing interest as a potential treatment for various cancers [22]. Investigation of the molecular mechanisms underlying hypericin photocytotoxicity in cancer cells have revealed that this photosensitizer can induce both apoptosis and necrosis in a concentration and light dose-dependent fashion [21, 23]. Moreover, PDT with hypericin results in the activation of multiple pathways that can either promote or counteract the cell death program [19]. Investigations of the molecular mechanisms underlying hypericin photocytotoxicity in cancer cells have revealed that this photosensitizer can induce apoptosis in a dose-dependent fashion. However, very soon after irradiation, JNK1 and p38 MAPK are activated. Inhibitor and transfection studies revealed that these responses increase the cellular resistance against hypericin-induced apoptosis in a caspase-independent manner, which allow the cells to cope with the damage caused by the insult [24]. In addition, hypericin also has been investigated as a powerful photosensitizer for inactivation of DNA and RNA viruses including human immunodeficiency virus (HIV), hepatitis C virus (HCV), and herpes simplex virus (HSV) [25–28]. However, the mechanisms by which photoactivated hypericin interferes with and inactivates viruses has been not clarified yet.

In this study, we investigated the efficacy of hypericin-PDT in ATL cells. We show that hypericin, in the context of PDT, inhibits the ATL cell growth by induction of apoptosis and suppression of viral transcription, indicating that hypericin is a promising drug for its characteristic of light-dependent antitumor and antiviral activity in ATL-targeted therapy.

## Results

### Photoactivated hypericin inhibits the proliferation of ATL cells

**Fig. 1:**
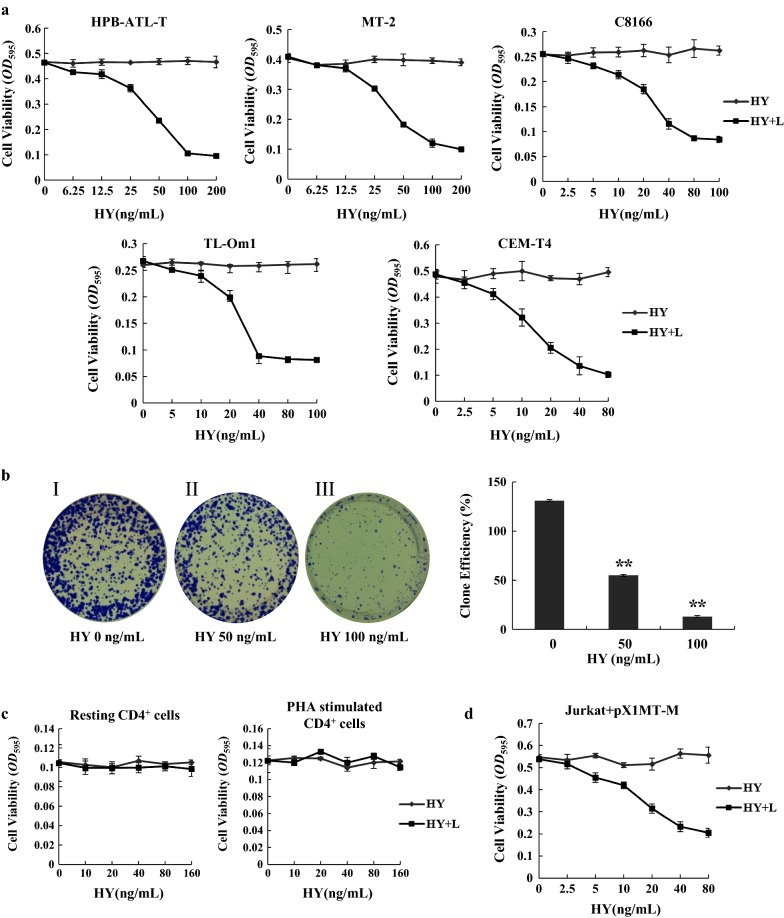
Hypericin-PDT induced growth arrest in ATL cells. **a** The effects of hypericin-PDT treatment on the growth of HTLV-1-positive cell lines (HPB-ATL-T, MT-2, C8166, and TL-Om1) and HTLV-1-negative T-cell line (CEM-T4). Cells were treated with increasing amounts of hypericin with or without light irradiation for 24 h. The proliferation of each cell was examined by methyl thiazolyl tetrazolium assay. HY indicates hypericin, and HY + L indicates hypericin with light irradiation, **b** influence of hypericin on colony forming efficiency of HPB-ATL-T cells. (Left panel) I: control group; II: 50 ng/mL hypericin-PDT group; III: 100 ng/mL hypericin-PDT group. (Right panel) Quantitative representation of colony forming efficiency on HPB-ATL-T cells, **c** resting and activated CD4^+^ T lymphocytes are resistant to hypericin-PDT. CD4^+^ T cells were isolated from PBMCs of healthy donor. Activated CD4^+^ T cells were supplemented with 10 ug/mL PHA. Cells were treated with hypericin with or without light irradiation up to 24 h. Cell growth was assayed in triplicate wells by MTT assay, **d** HTLV-1 infected Jurkat cells are sensitive to hypericin-PDT treatment. Jurkat were transfected with pX1MT-M by electroporation using Neon. Cells were treated with the indicated concentration of hypericin with or without light irradiation for 24 h. Cell growth was assayed by MTT assay. All statistical analyses are shown as *P < 0.05; **P < 0.01. Representative results of 3 independent experiments are shown

First, we analyzed the effect of hypericin on HTLV-1-associated T-cell lines (HPB-ATL-T, MT-2, C8166, and TL-Om1) and HTLV-1-negative cell line (CEM-T4) by MTT assay. Since the photosensitizing properties of hypericin are well established, we examined the effect of hypericin under light conditions (520–750 nm, 11.28 J/cm^2^). As shown in Fig. 1a, the treatment with hypericin and subsequent irradiation with visible light resulted in a dose-dependent growth inhibition of all tested cell lines, whereas hypericin alone had no effect. The half maximal inhibitory concentration (IC_50_) of hypericin-PDT against HPB-ATL-T, MT-2, C8166, TL-Om1, and CEM-T4 cell lines were 52.98 ± 10.11, 52.86 ± 10.57, 43.02 ± 9.25, 37.88 ± 9.36, and 19.04 ± 6.22 ng/mL, respectively. The number of ATL cells incorporated bromodeoxyuridine (BrdU) was decreased after the treatment of hypericin-PDT (Additional file 1: Figure S1). Similarly, the result of a colony-forming assay revealed that clonogenic survival of HPB-ATL-T cells was significantly decreased following hypericin-PDT treatment (Fig. 1b). In contrast, hypericin-PDT had no effect on resting and PHA-stimulated normal peripheral blood CD4^+^ T lymphocytes from healthy donors compared with ATL cells (Fig. 1c). As shown in Fig. 1d, hypericin-PDT treatment resulted in a growth inhibition of Jurkat cells which transfected with an infectious molecular clone of HTLV-1 (pX1MT-M). To study the effect of hypericin on HTLV-1 cell-to-cell transmission, we co-cultured hypericin-PDT treated HPB-ATL-T cells with WT-Luc transfected Jurkat cells. Luciferase assay revealed that hypericin-PDT treatment did not influence transmission of HTLV-1 from HPB-ATL-T to Jurkat cells (Additional file 1: Figure S2). Taken together, these results suggest that photoactivated hypericin effectively inhibits the proliferation of ATL cells.

### Hypericin-PDT induces apoptosis of ATL cells

**Fig. 2:**
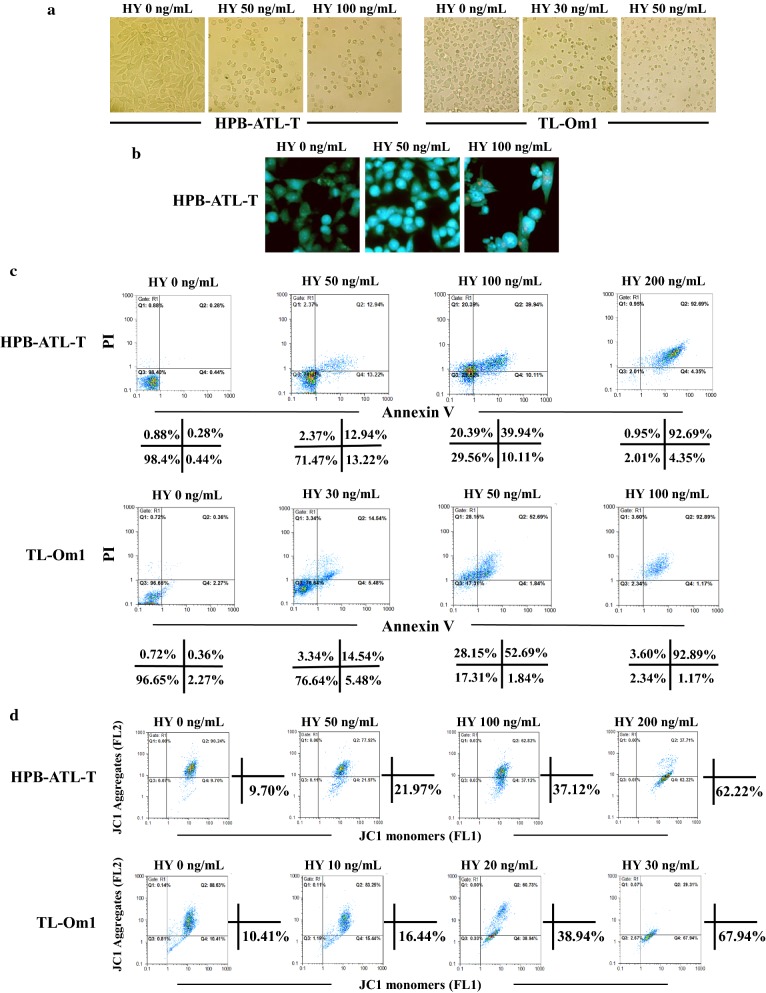
Hypericin-PDT induced apoptosis in ATL cells. **a** Hypericin-PDT apoptotic effects on cellular morphology of HPB-ATL-T and TL-Om1 cells by light microscopy. Cells were treated with increasing concentrations of hypericin for 16 h followed by irradiation for 30 min. The cells were further incubated for 24 h and cellular morphology was examined for each treatment, **b** apoptotic nuclei of HPB-ATL-T cells evaluated using fluorescence microscopy with AO (blue)/EB (red) double staining. **c** Representative flow-cytometric dot plot analysis of apoptosis after Annexin V-FITC/PI staining in HPB-ATL-T and TL-Om1 cells treated with hypericin-PDT. The percentages of each fraction are shown. Three major populations of cells can be observed in the cytotoxicity assay: Annexin V−/PI− cells are defined as live cells, Annexin V+/PI− cells as apoptotic cells and PI+ as dead cells (late apoptotic or necrotic), **d** effects of hypericin-PDT on changes of mitochondrial membrane potential (ΔΨm) in HPB-ATL-T and TL-Om1 cells. Representative flow cytometric dot plots in different groups. The percentages of JC-1 monomer are shown

To investigate the mechanisms involved in hypericin-PDT induced growth inhibition, we evaluated the effect of hypericin-PDT on cell apoptosis. After hypericin-PDT treatment, observation by microscopy showed that ATL cells display a typical morphological appearance of apoptosis, including cell shrinkage, membrane blebbing, chromatin condensation and fragments, compared to the untreated controls (Fig. 2a, b). Moreover, apoptotic bodies were formed in the 100 ng/mL hypericin-PDT-treated group. To further confirm the ability of hypericin-PDT to induce apoptosis of ATL cells, HPB-ATL-T and TL-Om1 cells were analyzed using Annexin V/PI double staining after the treatment of hypericin-PDT. Flow cytometry results showed that hypericin-PDT treatment resulted in a dose-dependent induction of cell apoptosis in both cell lines compared with the corresponding controls (Fig. 2c). It is well established that early apoptosis related to dissipation of mitochondrial membrane potential (ΔΨm) [29]. We therefore investigated whether hypericin-PDT could change mitochondrial membrane potential. As shown in Fig. 2d, treatment of hypericin-PDT increased the percentage of the JC-1 monomer (green fluorescence) in a dose-dependent manner, indicating that hypericin-PDT could induce the dissipation of ΔΨm.

Taken together, these observations demonstrated that the inhibitory effect on proliferation induced by hypericin-PDT was due to the promotion of apoptosis.

### Hypericin-PDT induces caspase dependent and independent apoptosis

**Fig. 3:**
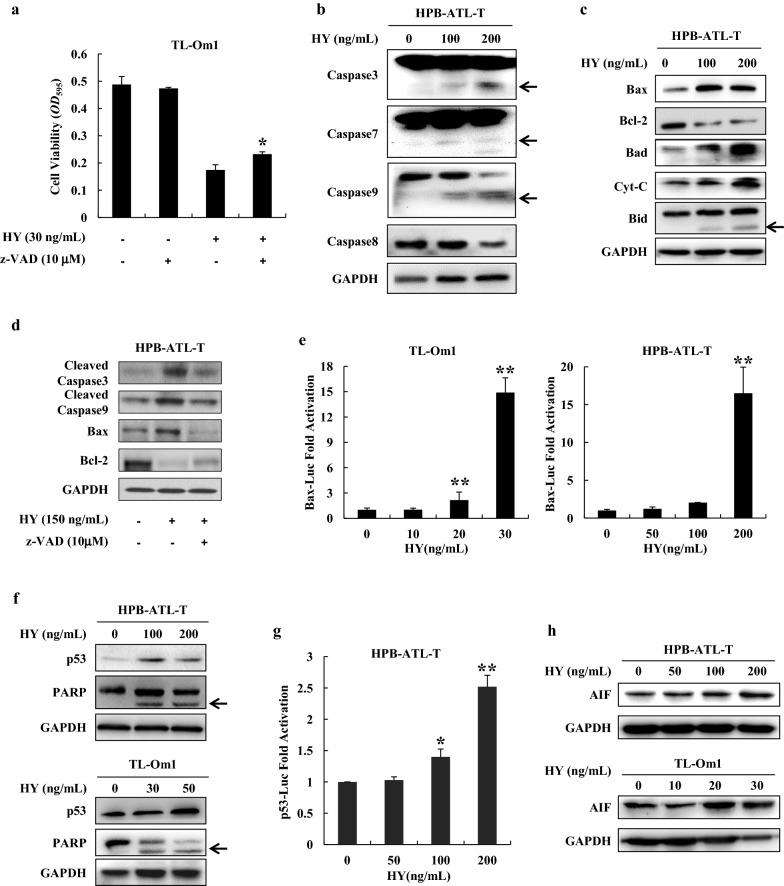
Hypericin-PDT induced caspase dependent and independent apoptosis. **a** Effects of the general caspase inhibitor z-VAD-fmk on hypericin-PDT induced growth inhibition of TL-Om1 cells. Cells were pretreated for 2 h with 10 μM z-VAD-fmk followed by 24 h treatment with 30 ng/mL hypericin. The proliferation of each cell was examined by MTT assay, **b** protein levels of caspase family proteins hypericin-PDT-treated cells. The expression of Caspase 3, Caspase 7, Caspase 8, and Caspase 9 were analyzed by immunoblotting. ← indicates cleavaged caspase protein, **c** hypericin-PDT treatment influenced the expression of apoptosis-related proteins. The expression of Bax, Bid, Bcl-2, Bad, and Cyt-C were analyzed by western blot. ← indicates cleavaged Bid protein, **d** z-VAD-fmk reversed the modulating function of hypericin-PDT on apoptosis-related proteins’ expression. HPB-ATL-T cells were pretreated for 2 h with 10 μM z-VAD-fmk followed by 24 h treatment with hypericin (150 ng/mL)-PDT. The expression of cleaved Caspase 3, cleaved Caspase 9, Bax, and Bcl-2 were analyzed by immunoblotting, **e** hypericin enhanced Bax promoter activity in TL-Om1 and HPB-ATL-T cells. Cells were transfected with the Bax reporter plasmid with or without hypericin-PDT treatment. Luciferase activity was measured 48 h after transfection, **f** western blot analysis for the expression of p53, PARP, and GAPDH proteins in hypericin-PDT-treated ATL cells. ← indicates cleavaged PARP protein, **g** hypericin-PDT activated p53 signaling pathway. Hypericin-PDT-treated HPB-ATL-T cells were cotransfected with p53-Luc and phRL-TK. After 48 h, the cells were harvested and analyzed for luciferase activity, **h** hypericin-PDT treatment influenced the expression of AIF protein. The expression of AIF and GAPDH were analyzed by western blot. Statistically significant differences are labeled *P < 0.05, **P < 0.01, compared with control group using Student’s *t*-test

To clarify the molecular mechanism by which hypericin-PDT inhibits the proliferation of ATL cells, we pretreated TL-Om1 cells with z-VAD-fmk, a pan-caspase inhibitor for 2 h prior to 24-h treatment with hypericin. Hypericin-PDT significantly inhibited cell proliferation in TL-Om1 cell line, and z-VAD-fmk was able to partially overcome growth inhibition caused by hypericin-PDT (*P* < 0.05) (Fig. 3a). Moreover, z-VAD-fmk restored hypericin mediated suppression of the percentage of ATL cells incorporating BrdU (Additional file 1: Figure S3). This indicates that hypericin-induced apoptosis of ATL cells is partially caspase-dependent. We next examined whether hypericin-PDT suppresses the proliferation of ATL cells through modulating the expression of caspase family proteins. As shown in Fig. 3b, cleaved caspase-3, caspase-7, and caspase-9 were increased in HPB-ATL-T cells with the treatment of hypericin-PDT, and the level of caspase-8 was downregulated. To assess whether hypericin-PDT is mediating the growth inhibitory effects through modulating the expression of other apoptosis-related proteins, we tested for several upstream and downstream caspase target proteins. As shown in Fig. 3c, the expression of Bax, Bad, and Cyt-C were upregulated, meanwhile the expression of Bcl-2 was downregulated after treatment with hypericin-PDT at 100, 200 ng/mL. Obviously, the ratio of Bax/Bcl-2 was significantly increased compared with the control. Cleaved Bid was clearly observed in hypericin-PDT-treated HPB-ATL-T cells. In addition, z-VAD-fmk reversed the dysregulation of hypericin-PDT on apoptosis-related proteins’ expression (Fig. 3d). To further analyze the mechanism by which hypericin-PDT induces Bax expression, we performed a reporter assay using the promoter of human *Bax* gene. As shown in Fig. 3e, Bax luciferase activity was increased nearly 16-fold by hypericin-PDT treatment when compared with untreated control. To further decipher hypericin-PDT mediated growth inhibition and cell death, p53 protein levels were monitored following hypericin-PDT treatment. As shown in Fig. 3f, hypericin-PDT induced a substantial upregulation of total p53 proteins. Indeed, the luciferase reporter assay revealed that hypericin-PDT activated p53 signaling occurs in a dose-dependent manner (Fig. 3g). In addition, the treatment with hypericin-PDT induced the caspase-3 mediated cleavage of the PARP protein in a dose-dependent manner (Fig. 3f). We next analyzed whether hypericin-PDT could also affect caspase-independent pathways. Expression of AIF protein was enhanced upon hypericin-PDT treatment in ATL cells (Fig. 3h). These results collectively indicate that an AIF-mediated caspase-independent apoptotic pathway and a Cyt-C mediated caspase-dependent apoptotic pathway are involved in hypericin-PDT induced cell death.

### Hypericin-PDT causes G2/M cell cycle arrest in ATL cells

**Fig. 4:**
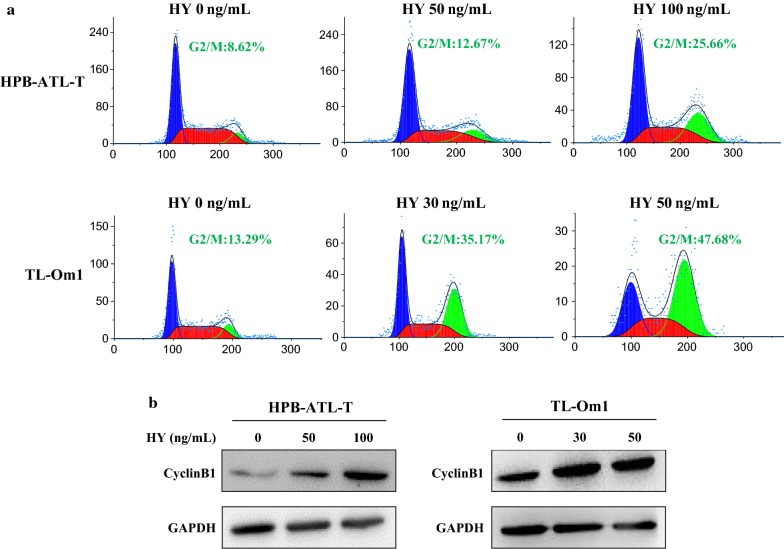
Hypericin-PDT induced G2/M cell cycle arrest in ATL cells. **a** Effects of hypericin-PDT on the cell cycle distribution of HPB-ATL-T and TL-Om1 cells. Cells were treated with hypericin-PDT up to 24 h and stained with PI (50 μg/mL) and the cell cycle analysis was performed using flow cytometry, **b** HPB-ATL-T and TL-Om1 cells were treated with hypericin-mediated PDT for 24 h. Hypericin-PDT regulates cyclinB1 protein expression as shown by western blot analysis. GAPDH was used as an internal control

To evaluate whether the hypericin-PDT induced growth inhibition and cell death was due to cell cycle arrest, we evaluated the cell cycle distribution of HTLV-1 positive cell lines after treatment with hypericin-PDT. Flow cytometry analysis of the cellular DNA contents revealed that hypericin-PDT significantly caused an arrest of ATL cells in the G2/M phase and decreased the percentage of cells in the S phase (Fig. 4a). To further elucidate the mechanism by which hypericin-PDT induced G2/M cell cycle arrest, western blot analysis was performed to check any changes in the levels of cyclinB1 protein. As shown in Fig. 4b, the expression of cyclinB1 was upregulated in all tested cells after treatment with hypericin-PDT. These results demonstrate that the inhibitory effect on proliferation induced by hypericin-PDT was partially due to the inhibition of cell cycle progression.

### Hypericin-PDT suppresses viral gene expression and viral transcription

**Fig. 5:**
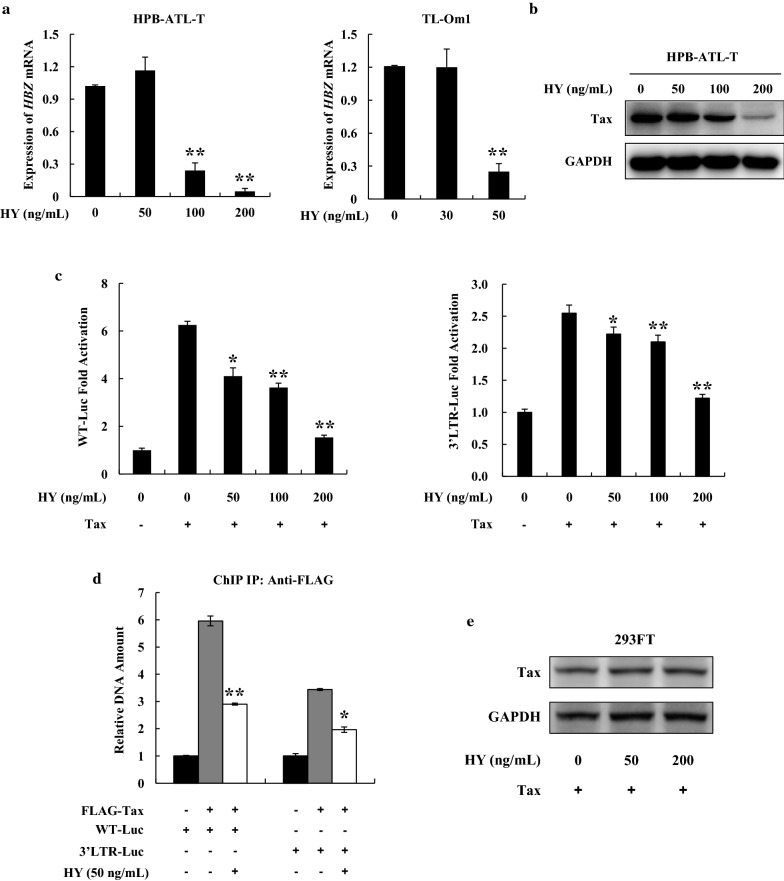
Hypericin-PDT suppressed the expression of viral genes and inhibited HTLV-1 LTR activity. **a** Expression of *HBZ* transcripts was detected by qPCR in HPB-ATL-T and TL-Om1 cells after treating with hypericin-PDT, **b** HPB-ATL-T cells were treated with hyerpicin-mediated PDT for 24 h. The expression of Tax and GAPDH were analyzed by western blot, **c** hypericin-PDT impaired the HTLV-1 5′LTR and 3′LTR promoter activity. Cells were transfected with the Tax and HTLV-1 LTR reporter plasmids. Luciferase activity was measured 48 h after hypericin-PDT treatment, **d** hypericin-PDT inhibited Tax DNA binding capability. 293FT cells were transfected with FLAG-Tax together with WT-Luc or 3′LTR-Luc. After hypericin-PDT treatment, 293FT cells were chromatin immunoprecipitated by anti-FLAG antibody. The precipitated DNAs and 1% of the input cell lysates were amplified using the WT-Luc or 3′LTR-Luc specific primers by real-time PCR. Statistically significant differences are labeled *P < 0.05, **P < 0.01, compared with control group using Student’s *t*-test

Accumulating evidence has shown that hypericin has potent antiretroviral activities [25, 26]. We therefore studied whether hypericin has any influence on HTLV-1. Expression of HTLV-1 encoded Tax protein and *HBZ* mRNA was analyzed by western blot and semiquantitative RT-PCR, respectively, after hypericin treatment. As shown in Fig. 5a, hypericin-PDT suppressed *HBZ* gene expression in a dose-dependent manner. The level of Tax protein was also downregulated in hypericin-PDT-treated HPB-ATL-T cells (Fig. 5b). To further analyze the mechanism by which hypericin-PDT inhibits viral gene expression, we performed a reporter assay using the HTLV-1 5′LTR (WT-Luc) and 3′LTR (3′LTR-Luc) promoter plasmids. Figure 5c illustrates that hypericin-PDT suppressed Tax mediated HTLV-1 5′LTR and 3′LTR promoter activation. To clarify the underlying mechanism, we performed a ChIP assay in 293FT cells that were cotransfected expression vector of Tax with WT-Luc or 3′LTR-Luc respectively. After hypericin-PDT treatment, the ChIP assay detected the association of Tax with its responsive elements in 5′LTR and 3′LTR of HTLV-1, while hypericin-PDT treatment dramatically decreased Tax’s DNA binding capability (Fig. 5d). However, hypericin did not induce Tax protein degradation even at high doses (Fig. 5e).

Taken together, the results suggest that hypericin-PDT impairs the HTLV-1 viral transcription through 5′LTR and 3′LTR, resulting in reduction of HBZ and Tax gene expression.

## Discussion

After the identification of adult T-cell leukemia (ATL) as a discrete clinical entity, much progress has been made in the treatment of the disease. Encouraging advances in treating ATL are coming from the effectiveness of hematopoietic stem cell transplantation and new molecular-targeted drugs [4, 6, 30]. However, current therapeutic regimens fail to improve the survival of ATL patients, stressing the need for alternative or complementary therapies. The ability of photodynamic therapy (PDT) to specifically recognize and destroy cancer cells is considered to be a milestone groundbreaking development [31]. In recent years, increased interest in hypericin (HY) as a potential clinical anti-tumor photosensitizer agent has arisen since several studies established its powerful antineoplastic and antiviral activities [19, 22]. Investigations of the molecular mechanisms underlying hypericin photocytotoxicity in cancer cells have revealed that this photosensitizer can induce both apoptosis and necrosis in a high-quantum yields and a low cytotoxicity fashion [21, 23]. It is demonstrated that the apoptosis induced by hypericin-mediated PDT is involved with plasma membrane death receptors, mitochondria, lysosomes and ER, caspases, and Bcl-2 family proteins [23]. Mitochondrial damage has been identified as one of the main events during PDT with hypericin [32]. Moreover, PDT with hypericin results in the activation of multiple pathways that can either promote or counteract the cell death program [33]. We are the first to present evidence that hypericin suppressed the proliferation of ATL cells mainly through mitochondrial dependent apoptotic signaling. In HTLV-1 infected cells, p53 checkpoint is ineffective in ATL; owing to Tax-mediated inactivation [34]. Our finding that hypericin-induced apoptosis of ATL cells is associated with the upregulation of p53 prompted us to investigate the involvement of p53 signaling activation in hypericin-induced apoptosis. Luciferase assay demonstrated that hypericin could enhance p53 pathway, indicating that p53-mediated apoptotic singling is essential for the treatment of ATL by hypericin-PDT. Further studies are necessary to clarify the mechanism by which hypericin enhances the expression of p53 in ATL cells.

It is well known that vigorously proliferating cells were more sensitive to natural product drugs or cytotoxic chemicals than nonproliferating/quiescent cells [35]. One possible reason for this is that most cytotoxic drugs targeted replicated DNA easily. Consistently, we showed in this study that hypericin-PDT treatment inhibited the growth of highly proliferating ATL cells, and suppressed DNA binding capability of HTLV-1 Tax protein. PBMCs which are nonproliferating cells don’t show any sensitivity to the photo-toxic effects of hypericin.

Accumulating evidence has shown that hypericin is a virucidal agent that effectively targets a broad range of enveloped viruses and retroviruses, including human immunodeficiency virus type 1 (HIV-l), hepatitis C virus (HCV), herpes simplex virus (HSV), influenza A (influenza virus type A), murine Friend leukemia virus, Rauscher leukemia virus, murine immunodeficiency virus, and duck hepatitis B virus (DHBV) [25–28]. Hypericin has been shown to associate with virions and their replication cycles [26, 28]. Meruelo et al. reported that hypericin prevents viral budding and shedding from cells by interfering with the proper assembly and maturation of viral cores of budding particles [34, 36]. Moreover, hypericin induced inactivation of HIV-1 is characterized by photochemical alteration of the HIV major capsid protein p24 and the p24-containing gag precursor, and it suppressed reverse transcriptase-activity from the treated virions [37]. The HCV genome encodes nonstructural protein 3 (NS3) helicase, which is essential for viral replication. Hypericin, which has hydroxyanthraquinone-like moieties, can inhibit NS3 helicase [38]. In vitro tests have shown that hypericin is effective in inactivating viruses endowed with lipid coats, while it is ineffective against non-enveloped viruses, suggesting that inactivation might depend on the presence of a viral lipid membrane [25]. HTLV-1 is an enveloped retrovirus that contains an outer envelope containing protruding viral glycoproteins and reverse transcriptase activity [1]. We have shown here that hypericin-PDT treatment suppresses the proliferation of HTLV-1 infected T cells. It is thus likely that hypericin-PDT may target envelope protein and reverse transcriptase of HTLV-1, and it suppressed viral transmission and viral genome replication.

HTLV-1 encodes Tax and HBZ genes, which regulate transcription from HTLV-1 long terminal repeats and various types of cellular promoters [39, 40]. Counteracting the function of Tax and HBZ might have prophylactic and therapeutic benefits. Treatment strategies targeting viral LTR and Tax protein has been reported [4, 6, 30]. Resveratrol, is a natural compound found in the skin of red grapes, abrogated Tax-mediated LTR transactivation. The administration of resveratrol also decreased the production of cell-free HTLV-1 virions. Treatment with resveratrol prevented the interaction of Tax with CREB and the recruitment of CREB, CRTC1, and p300 to Tax-responsive elements in the LTR [41]. In addition, a synthetic adamantly retinoid ST1926 induced growth inhibition in primary ATL cells and decreased the levels of Tax expression in vitro and in vivo. Hence, ST1926 is a promising synthetic retinoid in ATL-targeted therapy [42]. The present study indicates that HTLV-1 infected cells are sensitive to hypericin-PDT treatment, which may be due to hypericin-PDT-induced suppression of Tax and HBZ expression. Previous reports have shown that hypericin could inference the phosphorylation of CREB protein and dysregulate a variety of gene promoters including VEGF, GLUT1, IL-6, and IL-2 [43–45]. CREB plays a critical role in Tax-mediated HTLV-1 transcription by forming a complex with Tax that binds to viral cyclic AMP-response elements (CREs) located within the viral promoter [46]. Thus, we speculated that hypericin-PDT may also interfere the LTR activity of HTLV through dysregulating CREB, and lead to the suppression of Tax and HBZ expression. Our work reveals the interplay between hypericin and Tax, HBZ, thereby provides a new regulatory mechanism in HTLV-1 transcription, a control step in disease development. Our findings on hypericin may also guide future development of new anti-HTLV-1 agents for prevention and intervention against ATL and HAM/TSP.

Since all the blood cells, including leukemia cells, flow within a closed vascular system in the body and cannot be irradiated directly, many researchers have proposed to insert an optical fiber into the tail vein of an animal for the implementation of intravascular PDT [47]. Moreover, nontoxic and nonaggregating calcium phosphosilicate nanoparticles (CPSNPs) encapsulating the near-infrared fluoroprobe indocyanine green (ICG) were recently developed for diagnostic imaging and drug delivery as well as for PDT of solid tumors [48, 49]. ICG-loaded CPSNPs were evaluated as photosensitizers for PDT of leukemia [50]. Using a novel bioconjugation approach to specifically target CD117 or CD96, surface features enhanced on leukemia stem cells, in vitro ICG-CPSNP PDT of a human leukemia samples were dramatically improved [50]. Furthermore, the in vivo efficacy of PDT was dramatically enhanced in a murine leukemia model by utilizing CD117-targeted ICG-CPSNPs, resulting in 29% disease-free survival [50]. Thus, we believe that hypericin, with the application of in vivo photodynamic therapy, offers the promise to effectively treat ATL and to improve the life of leukemia patients.

## Conclusions

We showed that hypericin-mediated photodynamic therapy (PDT) suppressed the proliferation of ATL cells mainly through mitochondrial dependent apoptotic signaling. Moreover, hypericin-PDT impaired the HTLV-1 viral transcription through 5′LTR and 3′LTR, resulting in reduction of HBZ and Tax gene expression.

## Methods

### Cell culture

HTLV-1-infected T-cell lines (HPB-ATL-T, MT-2, C8166, and TL-Om1), and HTLV-1-negative T-cell lines (CEM-T4 and Jurkat) were cultured in RPMI 1640 supplemented with 10% FBS and antibiotics. 293FT cells were cultured in Dulbecco modified Eagle medium (DMEM) supplemented with 10% FBS and 500 μg/mL G418. Human peripheral blood mononuclear cells (PBMCs) were isolated from healthy HTLV-1-negative donors. CD4^+^ T cells were isolated by human naive CD4 T-cell enrichment set (BD Biosciences) according to the manufacturer’s instructions. To obtain activated CD4^+^ T cells, CD4^+^ T cells were stimulated by 10 μg/mL phytohemagglutinin (PHA) (Sigma-Aldrich, St. Louis, MO, USA).

### Drugs

A stock solution of 1 mg/mL hypericin (Santa Cruz, CA, USA) was prepared with dimethyl sulfoxide (DMSO; Sigma-Aldrich, St Louis, MO). The working solution of hypericin was diluted in RPMI1640 medium without serum. The final concentrations of DMSO never exceeded 0.1%, which showed no effect on the proliferation of all tested cells. z-VAD-fmk caspase inhibitor was purchased from Selleck Chemicals (Houston, TX).

### The 3-(4, 5-dimethylthiazol-2-yl)-2, 5-diphenyltetrazolium bromide (MTT) assay

Cells were treated with indicated concentrations of hypericin for 16 h in the dark. Subsequently, the cells were illuminated under the lighting conditions (520–750 nm, 11.28 J/cm^2^) for 30 min. Following irradiation, the cells were further incubated for 24 h in the dark. Cell growth were assessed by measuring the 3-(4, 5-dimethylthiazol-2-yl)-2, 5-diphenyl tetrazolium bromide (MTT) dye absorbance of the cells. Jurkat were transfected with pX1MT-M plasmid by electroporation using Neon (Life technologies). Cells were treated with the indicated concentration of hypericin with or without light irradiation for 24 h. Cell growth was analyzed by MTT assay.

### BrdU staining

Cells were labeled with 10 μM BrdU in culture medium for 30 min. After trypsinization and PBS wash, BrdU incorporation in ATL cells was detected using FITC BrdU Flow Kits (BD Pharmingen) according to the manufacturer’s instructions.

### Colony formation assay

HPB-ATL-T cells were treated in line with the experimental design. HPB-ATL-T cells were plated on 6-well plates at 300 cells per well, and kept in complete medium for 12 days. Colonies were fixed with methanol and stained with methylene blue. Colony formation was determined by counting the number of stained colonies. Triplicate wells were measured in each group.

### Light and fluorescence microscopy

After hypericin-PDT treatment, cells were collected for the following morphological analysis. Some cells were collected for acridine orange/ethidium bromide (AO/EB) co-staining, then examined under a fluorescence confocal microscopy (Leica TCS SP5 AOBS).

### Measurement of apoptotic cell death and cell cycle analysis

For detection of apoptosis, the Annexin V-binding capacities of the treated cells were examined by flow cytometry using an Annexin V-FITC Apoptosis Detection Kit (MultiSciences, Zhejiang, China). Cell cycle analysis was performed using propidium iodide (PI) (50 μg/mL) (Sigma) staining and a FACS scan flow cytometer (Partec, Berlin, German).

### Immunoblotting

Cell lysates were collected and subjected to standard Western blot analysis as described previously with antibodies against caspase-3, -7, -8, -9, Bax, Bcl-2, Bad, Cyt-C, Bid, p53, PARP, AIF, CyclinB1, Tax, and GAPDH (Cell Signaling Technology, Beverly, MA, USA).

### Luciferase assay

Jurkat cells were transfected with the indicated plasmids using Lipofectamin LTX Reagent (Invitrogen). Luciferase activity was measured 48 h after transfection using the Dual-Luciferase Reporter Assay System (Promega, Madison, WI).

### The 5, 5′, 6, 6′-tetrachloro-1, 1′, 3, 3′-tetraethylbenzimidazolyl-carbocyanine iodide (JC-1) staining

The collapse of the mitochondrial membrane was measured using a mitochondrial membrane potential assay kit with JC-1 (Beyotime Institute of Biotechnology, China). After JC-1 staining, cells were analyzed by flow cytometry.

### Quantitative real-time PCR

Total RNA was isolated using Trizol Reagent (Invitrogen, Carlsbad, CA, USA). Complementary DNA was synthesized using the SuperScript III reverse transcriptase (Life Technologies, Grand Island, NY, USA). Quantitative PCR (qPCR) was carried out using Power SYBR Green PCR Master Mix and StepOnePlus Real Time PCR System (Thermo Fisher Scientific, Waltham, MA, USA). Sequences for the primer set can be found in Additional file 1: Table S1.

### Chromatin immunoprecipitation assay

293FT cells were transfected with the indicated plasmids. At 48 h after transfection, a ChIP assay was done according to the protocol recommended by Upstate Biotechnology. Precipitated DNA was amplified by PCR using primers specific for the WT-Luc or 3′LTR-Luc respectively. Sequences for the primer set can be found in Additional file 1: Table S1.

### HTLV-1 transmission assay

HPB-ATL-T cells were treated with 50 ng/mL hypericin in association with PDT. After 16 h, HPB-ATL-T cells were pretreated with 10 μg/mL mitomycin C (MMC) (Sigma-Aldrich) for 1 h. HTLV-1 transmission assay was performed by coculturing WT-Luc transfected Jurkat cells with hypericin-PDT treated HPB-ATL-T cells at a 2.5:1 ratio for 36 h. Jurkat cells were harvested for a dual-luciferase assay, and supernatant was collected for detecting HTLV-1 p19 protein using HTLV p19 Antigen ELISA Kit (Zeptometrix, Buffalo, NY).

### Statistical analyses

Statistical analyses were performed using the unpaired Student *t* test.

## Additional file

**Additional file 1: Figure S1.** Hypericin-PDT suppressed proliferation of ATL cells. Hypericin-PDT treated HTLV-1-positive cell lines (HPB-ATL-T, MT-2, and C8166) were labeled with 10 μM BrdU in culture medium for 30 min. After trypsinization and PBS wash, BrdU incorporation in ATL cells was detected using FITC BrdU Flow Kits (BD Pharmingen). Statistically significant differences are labeled *P < 0.05, **P < 0.01, compared with control group using Student’s *t*-test. **Figure S2.** Hypericin-PDT did not influence cell-to-cell transmission of HTLV-1. WT-Luc transfected Jurkat cells were mixed at a 2.5:1 ratio with hypericin-PDT treated HPB-ATL-T cells. After 36 h, Jurkat cells were collected and luciferase assay was performed (left panel). Levels of HTLV-1 p19 antigen were determined in cell culture supernatants by ELISA (right panel). **Figure S3.** z-VAD-fmk restored growth inhibition caused by hypericin-PDT using BrdU incorporation assay. TL-Om1 cells were pretreated for 2 h with 10 μM z-VAD-fmk followed by 24 h treatment with 30 ng/mL hypericin. Cells were labeled with 10 μM BrdU in culture medium for 30 min. After trypsinization and PBS wash, BrdU incorporation in ATL cells was detected using FITC BrdU Flow Kits (BD Pharmingen). Statistically significant differences are labeled *P < 0.05, **P < 0.01, compared with control group using Student’s *t*-test. **Table S1.** List of primers for quantitative PCR and chromatin immunoprecipitation.
