# Supplementary material for: Hypericin-photodynamic therapy inhibits the growth of adult T-cell leukemia cells through induction of apoptosis and suppression of viral transcription
Source: Retrovirology. 2019 Feb 19;16:5. doi: 10.1186/s12977-019-0467-0 (PMC6381730; doi:10.1186/s12977-019-0467-0)
Supplement: Supplementary file 1 — Additional file 1: Figure S1. Hypericin-PDT suppressed proliferation of ATL cells. Hypericin-PDT treated HTLV-1-positive cell lines (HPB-ATL-T, MT-2, and C8166) were labeled with 10 μM BrdU in culture medium for 30 min. After trypsinization and PBS wash, BrdU incorporation in ATL cells was detected using FITC BrdU Flow Kits (BD Pharmingen). Statistically significant differences are labeled *P < 0.05, **P < 0.01, compared with control group using Student’s t-test. Figure S2. Hypericin-PDT did not influence cell-to-cell transmission of HTLV-1. WT-Luc transfected Jurkat cells were mixed at a 2.5:1 ratio with hypericin-PDT treated HPB-ATL-T cells. After 36 h, Jurkat cells were collected and luciferase assay was performed (left panel). Levels of HTLV-1 p19 antigen were determined in cell culture supernatants by ELISA (right panel). Figure S3. z-VAD-fmk restored growth inhibition caused by hypericin-PDT using BrdU incorporation assay. TL-Om1 cells were pretreated for 2 h with 10 μM z-VAD-fmk followed by 24 h treatment with 30 ng/mL hypericin. Cells were labeled with 10 μM BrdU in culture medium for 30 min. After trypsinization and PBS wash, BrdU incorporation in ATL cells was detected using FITC BrdU Flow Kits (BD Pharmingen). Statistically significant differences are labeled *P < 0.05, **P < 0.01, compared with control group using Student’s t-test. Table S1. List of primers for quantitative PCR and chromatin immunoprecipitation. [file 12977_2019_467_MOESM1_ESM.pptx]

## Slide 1
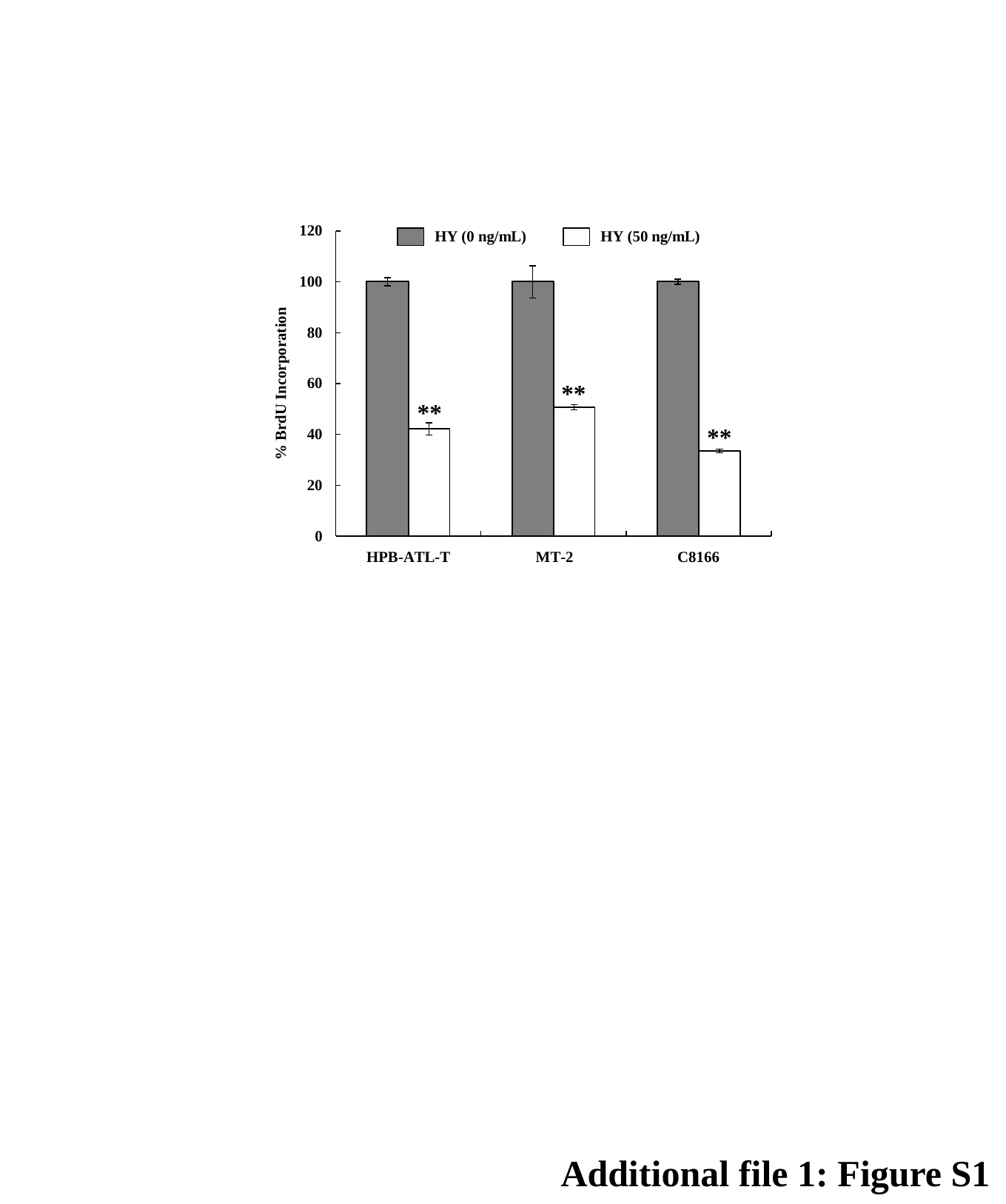

Additional file 1: Figure S1

## Slide 2
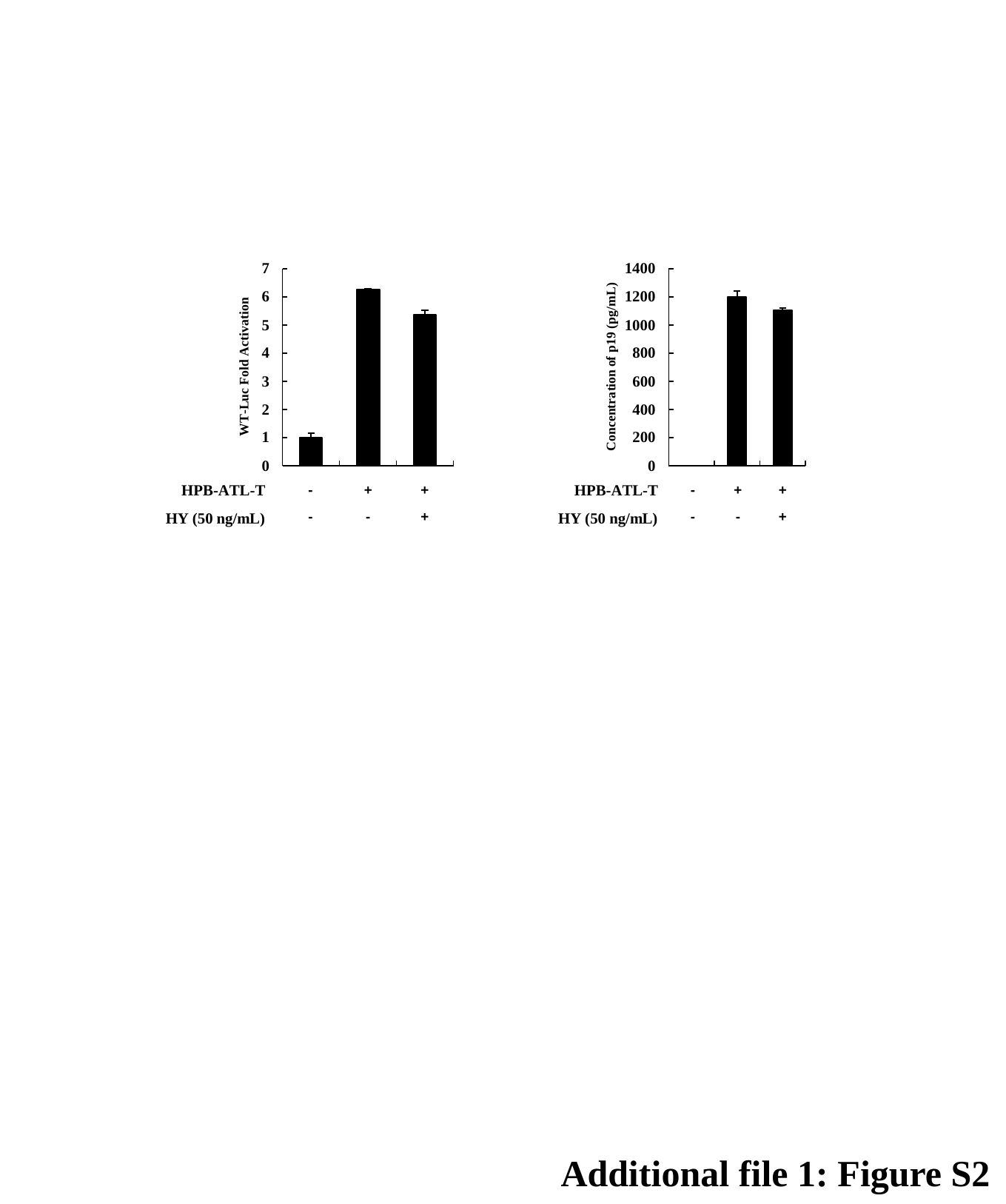

Additional file 1: Figure S2

## Slide 3
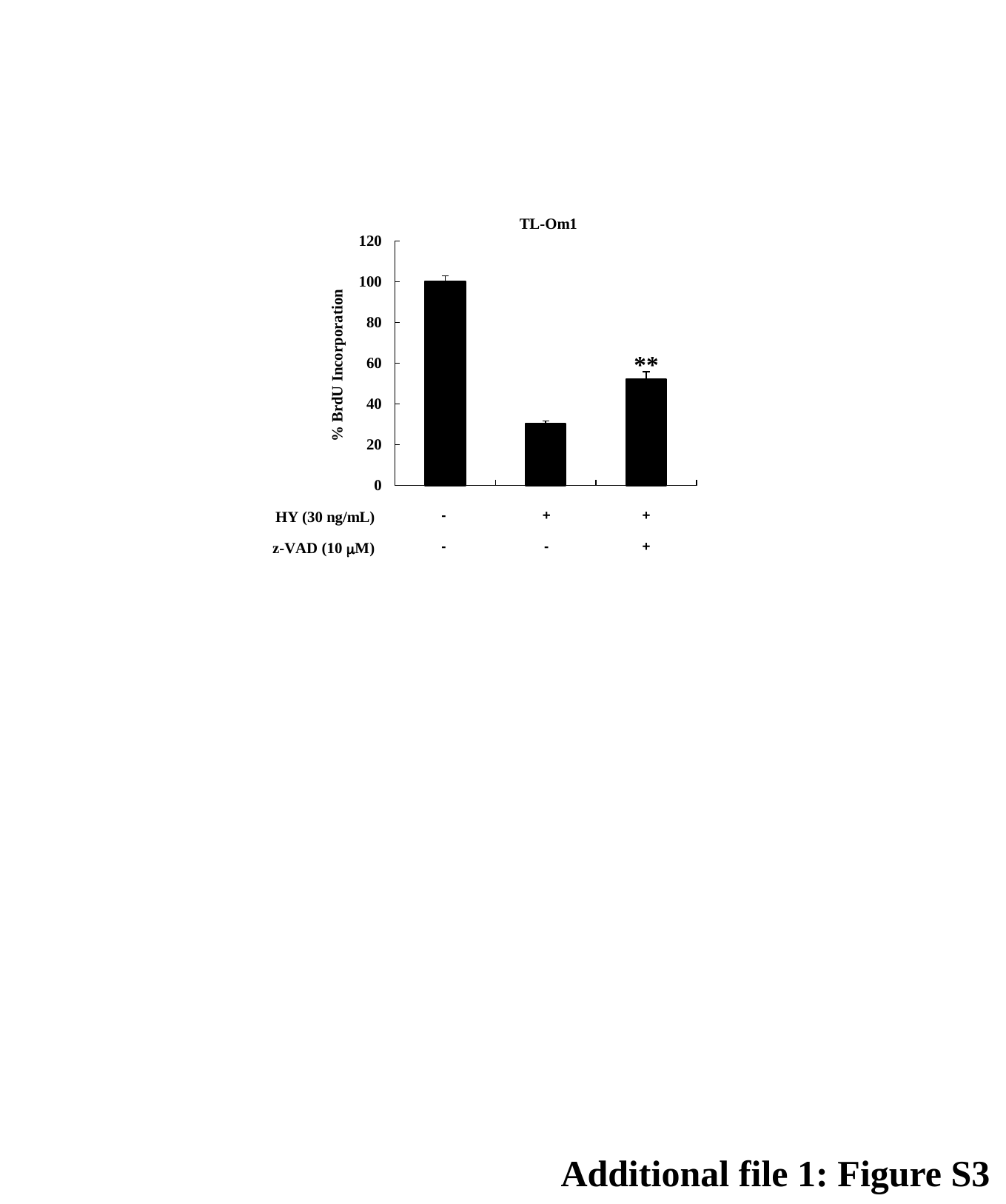

Additional file 1: Figure S3

## Slide 4
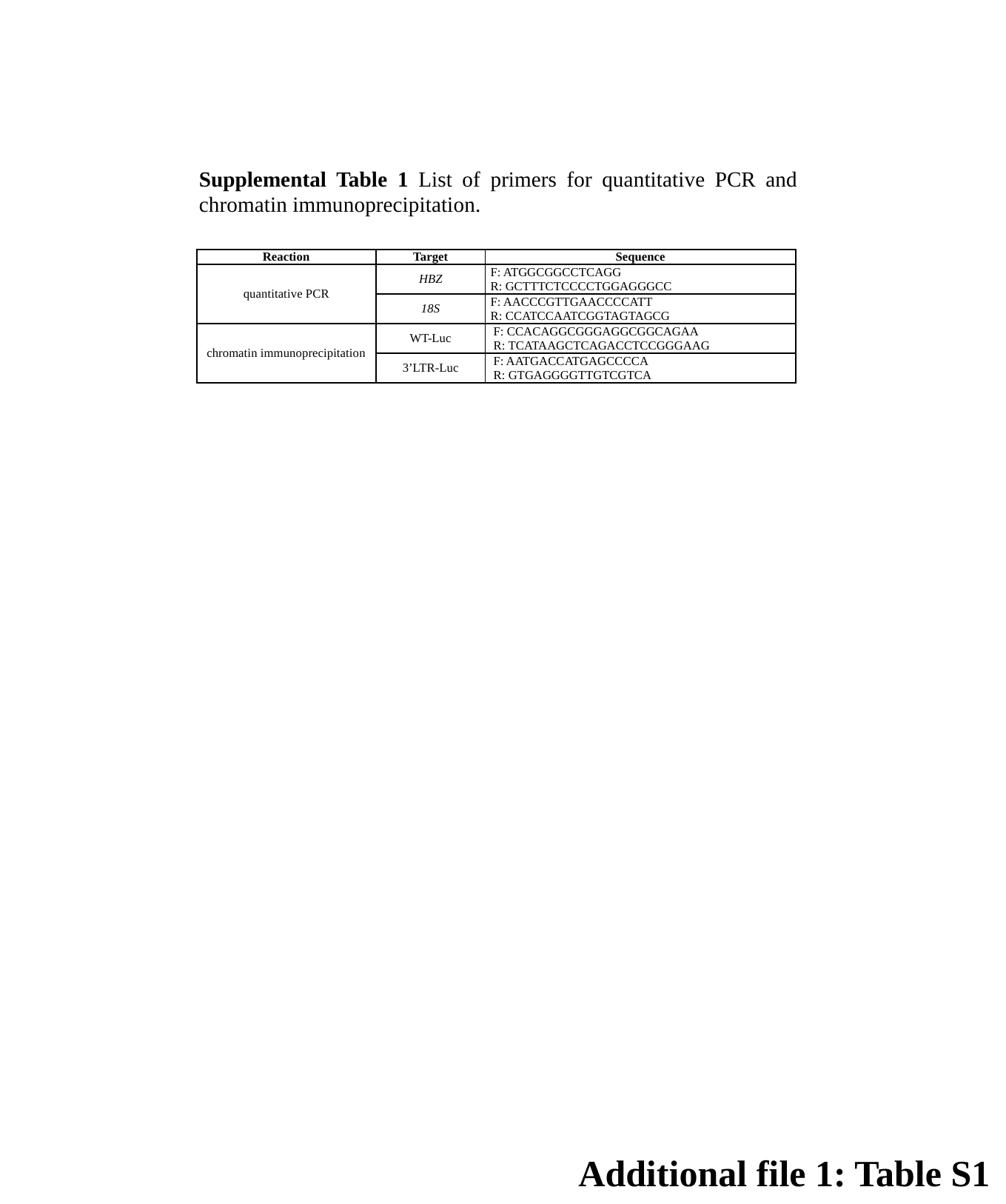

Supplemental Table 1 List of primers for quantitative PCR and chromatin immunoprecipitation.
| Reaction | Target | Sequence |
| --- | --- | --- |
| quantitative PCR | HBZ | F: ATGGCGGCCTCAGG R: GCTTTCTCCCCTGGAGGGCC |
| | 18S | F: AACCCGTTGAACCCCATT R: CCATCCAATCGGTAGTAGCG |
| chromatin immunoprecipitation | WT-Luc | F: CCACAGGCGGGAGGCGGCAGAA R: TCATAAGCTCAGACCTCCGGGAAG |
| | 3’LTR-Luc | F: AATGACCATGAGCCCCA R: GTGAGGGGTTGTCGTCA |
Additional file 1: Table S1
